# Success Rates of a CAD/CAM Nickel–Titanium Orthodontic Fixed Retainer

**DOI:** 10.3390/jcm14248762

**Published:** 2025-12-11

**Authors:** Luis Huanca Ghislanzoni, Candice Durgnat, Gregory S. Antonarakis

**Affiliations:** Division of orthodontics, University Clinics of Dental Medicine, University of Geneva, 1205 Geneva, Switzerland; luis.huanca@unige.ch (L.H.G.); gregory.antonarakis@unige.ch (G.S.A.)

**Keywords:** CAD/CAM, retainer, retrospective study, fixed retainer

## Abstract

**Background/Objectives:** The present study aims to assess the success rate of a CAD/CAM nickel–titanium wire (Memotain^®^) used as a fixed orthodontic retainer, over a one-year period. **Methods:** A retrospective study was conducted on 338 CAD/CAM nickel–titanium (Memotain^®^) fixed retention wires in 205 patients, bonded by a single experienced operator between January 2017 and December 2020. Follow-up visits were scheduled 6 (T1) and 12 months (T2) post-bonding. At each follow-up visit, events (defined as debonding, breakage, retainer loss, or tooth displacement) were classified by tooth, and success or failure of the retainer was determined based on the presence or absence of these events. **Results:** For the mandibular arch at T1 (6 months), the success rate was 85%, with debonding (*n* = 46) being the only event observed. At T2 (12 months), the success rate was 77%, with debonding (*n* = 30), wire breakage (*n* = 5) and retainer loss (*n* = 18) having occurred. For the maxillary arch, the overall success rate was 83% at T1 and 78% at T2. Debonding was the most common event observed over the 12-month observation period (*n* = 29), followed by retainer loss (*n* = 20) and wire breakage (*n* = 3). The overall success rates per type of tooth in the upper arch were 86% for the premolars, 96% for the canines, 95% for the lateral incisors and 93% for the central incisors. For the mandibular arch the success rates were 92% for the premolars, 97% for the canines, 96% for the lateral incisors and 94% for the central incisors. **Conclusions:** CAD/CAM nickel–titanium fixed retainers (Memotain^®^) demonstrated promising 1-year survival rates in both arches, though long-term multicentre studies are needed to confirm their reliability.

## 1. Introduction

Orthodontic retention is an integral part of orthodontic treatment, with fixed retainers being in widespread use in many different countries and health systems [1]. Without adequate retention, orthodontic treatment outcomes tend to relapse; however, across retainer types and strategies, the certainty of evidence regarding retainer selection remains low to very low at 12 months, according to a Cochrane review from 2023 [2]. In Switzerland for example, 87% of orthodontists recommend a lifelong retention with bonded retainers [3]. With a considerable number of patients undergoing orthodontic treatment and terminating treatment with the bonding of fixed retainers, survival of these retainers is crucial in avoiding frequent emergency appointments following bonding failure of these fixed retainers. By bonding fixed retainers, one needs to accept the fact that long-term maintenance is required, and this may be a shared responsibility among the patient, general dentist, dental hygienist and orthodontist.

The prevalence of failure of fixed orthodontic bonded retainers has been found to be 35% based on recent meta-analysis data [4], and this seems to increase with the duration of follow up. Long-term follow-up data show a 54% failure rate [4]. Failure can be defined in several ways, and this can include fracture of the wire or debonding of the composite which can include partial or total loosening of the retainer from the teeth, but also total retainer loss. The most common side effect is debonding of a point of composite or wire breakage. Although prolonged fixed retainer use may be effective in maintaining tooth position [2], inadvertent tooth movements also sometimes referred to as “wire syndrome” can occur with the use of multi-stranded fixed retention wires [5].

As an alternative to multi-stranded fixed retainers, avoiding the potential problems of inadvertent tooth movements, computer-aided design and manufacturing (CAD/CAM) fabricated nickel–titanium (NiTi) wire have been introduced, marketed, for example, as Memotain^®^ (CA-Digital, Mettmann, Germany) [6]. These allow fabrication of nickel–titanium (NiTi) lingual retainers that are individually designed from digital impressions and laser-cut with high precision. CAD/CAM-fabricated lingual retainers can be positioned with high accuracy relative to the planned virtual setup, supporting the rationale for custom-made devices [7]. Moreover, clinical findings have shown that flat and uniform bonding surfaces can be achieved across all teeth, with less need for intraoral adaptation, highlighting the precision of CAD/CAM retainer fit [7].

Beyond clinical precision placement, several studies have highlighted additional advantages of bonded CAD/CAM retainers. Knaup et al. [8] demonstrated that CAD/CAM NiTi retainers exhibit superior oral health parameters and significantly less biofilm formation compared to conventional multi-stranded retainers. Roser et al. [9,10] showed that NiTi CAD/CAM retainers produce significantly fewer artefacts on magnetic resonance imaging than traditional multi-stranded wires, offering a notable diagnostic advantage in head and neck imaging. Furthermore, Roser et al. [11] reported that NiTi CAD/CAM retainers restrict horizontal tooth mobility significantly less than other CAD/CAM materials such as zirconia or cobalt chromium, thereby better preserving physiological mobility.

To date, there have been a handful of clinical studies looking into failure of these CAD/CAM NiTi retention wires. Jowett et al. [12] in a randomized controlled trial found that of the 26 patients where CAD/CAM NiTi fixed retainers were placed, 50% of maxillary and 35% of mandibular retainers failed at the 6-month follow-up and thus the trial was prematurely terminated due to the high failure rates. Kartal et al. [13] found a 23% failure rate with CAD/CAM NiTi wires during a 6-month follow-up period in 26 patients, which was no different to that of a more traditional five-stranded bonded retainer. In a one-year follow-up, Gelin et al. [14] found a roughly 36% failure rate for CAD/CAM NiTi retention wires in 28 patients which was comparable to stainless steel retainers.

Two two-year follow-ups of a randomized controlled trials have been carried out, where one found that CAD/CAM NiTi fixed retainers in 90 patients showed 34% failure in the maxilla and 42% failure in the mandible [15], while the other study found that these retainers in 14 patients showed 21% failure [16], which was once again comparable to conventional fixed retainers. A three-year follow-up of a randomized clinical trial revealed that 8% of the 24 patients who had CAD/CAM NiTi retention wires showed failure, which was no different to when using multi-stranded stainless-steel wires [17].

Despite several prospective clinical trials evaluating the performance of CAD/CAM NiTi retention wires, real-world evidence remains limited. In particular, little is known about how these retainers fail in everyday clinical practice, including the distribution of failures by tooth type and the specific nature of each adverse event.

Therefore, the aim of the present retrospective study was to assess the failure rate of CAD/CAM NiTi maxillary and mandibular fixed retainers in a real-world clinical setting, and to analyse the number, type, and location of adverse events occurring approximately 6 and 12 months after bonding.

## 2. Materials and Methods

### 2.1. Material

**Figure 1:**
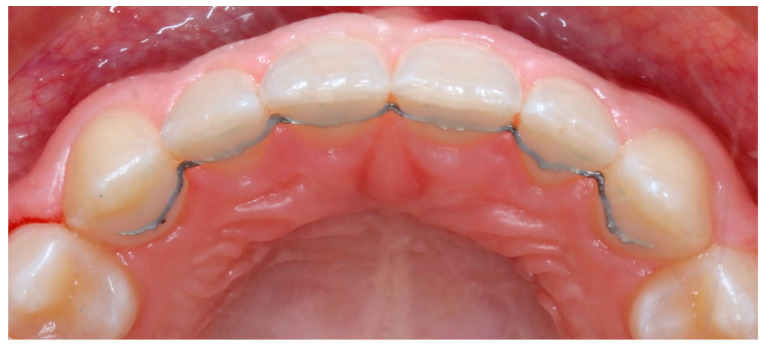
Picture of a 3 + 3 Memotain^®^ fixed retention wire after bonding.

The present investigation was a retrospective cohort study. Consecutive patients who received CAD/CAM nickel–titanium (NiTi) fixed retainers (Memotain^®^, CA-Digital, Mettmann, Germany) between January 2017 and December 2020 were screened for eligibility. Patients were eligible for inclusion if they had at least one Memotain^®^ fixed retainer bonded by a single experienced orthodontist (LH) at the end of active orthodontic treatment (Figure 1) and attended scheduled retention follow-up visits at approximately 6 months (T1) and 12 months (T2) after debonding.

Exclusion criteria were: the use of any other type of fixed retainer; bonding performed by a different operator; tooth agenesis, extraction, enamel developmental defects, or restorations (e.g., crowns, large composite build-ups) of any tooth where the fixed retainer was to be bonded; a patient not showing up for either the 6- or 12-month follow-up appointment. However, a certain amount of leeway was allowed for the 12-month follow-up visit, whereby an appointment up to 18 months following debonding was deemed acceptable.

All retainers were bonded by a single experienced operator (LH) following a standardized protocol. The teeth to be bonded were cleaned using a fluoride-free polishing paste and isolated using a Nola Dry Field System (Great Lakes Dental Technologies, ST. Ann, MO, USA) retractors. The lingual enamel surfaces were etched with 37% orthophosphoric acid for 30 s, rinsed thoroughly with water, and air-dried for 20 s.

A thin layer of Transbond XT primer (3M Unitek, Monrovia, CA, USA) was applied and light-cured for 20 s. The Memotain^®^ retainer was then positioned intraorally using the manufacturer-provided transfer key, and a flowable composite (Transbond Supreme LV, 3M Unitek, Monrovia, CA, USA) was placed over each contact point between the retainer and the tooth surface. Each bonding site was light-cured for 30 s.

Retainers were bonded from lateral incisor to lateral incisor, canine to canine, or premolar to premolar, depending on the initial malocclusion and treatment objectives for each patient. No removable retainers were prescribed in conjunction with the fixed bonded retainers.

The follow-up protocol of the operator was a follow-up retention check-up at approximatively 6 months post debonding (T1) and fixed retainer bonding, and a subsequent one at approximatively 12 months post debonding (T2). In case of emergency, the patients were instructed to schedule an appointment at their earliest convenience. At each of these appointments, the operator noted any failure, indicating the exact tooth where failure was present if composite detachment had occurred, or the location of fracture if wire fracture had occurred.

### 2.2. Methods

A chart review was carried out looking at the debonding appointment, the T1 and T2 follow-up appointments, and any intermediate emergency appointments. For each bonded wire, the date of application and the teeth to which it was bonded were noted. From the T1 and T2 follow-up appointments, or any emergency appointments, events were recorded accompanied with the date of the follow-up appointment. The events recorded were classified in four distinct but not mutually exclusive categories: debonding (including tooth or teeth in question), wire breakage (including location), wire loss, and tooth displacement. If no event took place, then this was also recorded and defined as a success. In the case where both maxillary and mandibular bonded retainers were in place, this was done individually for each bonded retainer. Negative events were defined as any adverse occurrence affecting the fixed retainer, including debonding, wire breakage, wire loss, or tooth displacement. For the survival analysis, failures were defined as wire breakage or complete wire loss, while simple debonding without wire fracture was not considered a failure. All data were extracted and recorded anonymously.

### 2.3. Statistical Analysis

Differences among groups were presented descriptively in tables, showing absolute counts and corresponding percentages for each category. The Kaplan–Meier estimator was used for the survival of the fixed retainers. All statistical analyses were performed using R version 2023.12.0.

## 3. Results

### 3.1. Sample

Over the four years of patient inclusion, 437 patients were bonded with Memotain^®^ fixed retainers, totalling 720 bonded Memotain^®^ wires. Subsequent to the application of the eligibility criteria, 232 patients were excluded, leaving a total of 205 included patients with 338 bonded Memotain^®^ fixed retainers (Table 1). From the 205 patients, 133 had a Memotain^®^ retention wire bonded on both arches, while 72 had a Memotain^®^ retention wire bonded on only one arch. From the total of 338 Memotain^®^ fixed retention wires, 162 (48%) were bonded on the maxillary arch and 176 (52%) on the mandibular arch. The scheduled 6- and 12-month follow-up appointments were carried out at 5.8 ± 1.9 months (T1) and 12.8 ± 2.2 months (T2).

### 3.2. Mandibular Fixed Retainers

During the first six months of follow up (T1), 26 of the 176 mandibular retainers had a negative event (15%), with the only event recorded being debonding. No wire breakage, loss, or tooth displacement was observed. Looking at tooth types, central and lateral incisors seemed to debond most often (5.1%) followed by canines (2.8%) (Table 2).

After twelve months of follow-up (T2), 26 of the 176 mandibular retainers had a negative event (15%) from the 6th to the 12th month. Events that had occurred were debonding, wire breakage or wire loss (Table 3). No tooth displacement (relapse of tooth position) was observed. A closer look at each tooth type reveals more negative events on the premolars (20%), followed by the central incisors (6.8%), the lateral incisors (3.4%) and finally the canines (2.5%) (Table 3).

From the retention wires with negative events at T2, 12 of the 26 already had a negative event observed at T1. This means that 46% of the negative events in the second half of the observation period occurred on retention wires that had already experienced a prior negative event.

An overall one-year success rate can be calculated by considering the retention wires that had negative events at T1, at T2 and those where negative events were observed at both T1 and T2. There were 12 retention wires with negative events observed at both T1 and T2, plus 14 that had a recorded negative event only at T1 and 14 with a recorded negative event only at T2. Therefore, a total of 40 retention wires showed negative events at the one-year follow-up, with an observed overall success rate of 77.3%. The overall success rates per type of tooth in the mandible were 92% for the premolars, 97% for the canines, 96% for the lateral incisors and 94% for the central incisors.

### 3.3. Maxillary Fixed Retainers

For the maxillary arch, at T1, 28 of the 162 retention wires had a negative event (17%). Events recorded were debonding (3.8%), tooth displacement (1.2%), wire loss (0.5%), wire breakage (0.2%). A closer look at each tooth reveals more negative events on the premolars (6%; although only 16 premolars were bonded) and canines (6%), than on the central and lateral incisors (5%) (Table 4).

Between the T1 and T2 follow-up appointments, 20 fixed retainers encountered a negative event (12%). There was no tooth displacement during this period. The most common negative events were wire loss (4.7%) followed by debonding (3.8%), and finally wire breakage (0.3%). When encountering debonding, the most affected teeth were the central (5.2%) and lateral (2.8%) incisors (Table 5).

Over the whole follow-up period, out of the 162 maxillary retention wires placed, 35 had a negative event at some time point (T1, T2, or both), which implies a failure rate of 22% (success rate 78%) at one-year follow up. Looking at the maxillary arch at T1 and T2, of the 28 retention wires that had a negative event at T1, 13 of these (65%) also had a negative event at T2. The most common negative events encountered in the maxillary arch over the whole follow-up period were debonding, especially for the central (5.8%) and lateral (2.8%) incisors, followed by wire loss. Based on these data, the overall success rates per type of tooth in the maxillary arch was 86% for the premolars, 96% for the canines, 95% for the lateral incisors and 93% for the central incisors.

### 3.4. Fixed Retainer Failure

**Figure 2:**
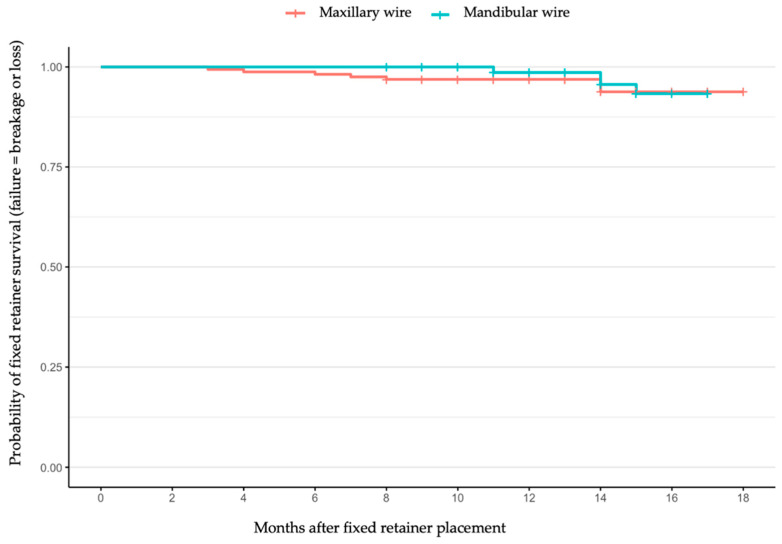
Kaplan–Meier survival plot for the maxillary and mandibular retainers up to 18 months after placement.

Using the Kaplan–Meier estimator for the survival of the retention wires (considering survival as the retention wire being maintained in place without breakage or loss), the survival is shown in the Kaplan–Meier survival plot in Figure 2. Retainers with no failure at the last recorded visit (maximum 18 months) were censored at that time. As the study was descriptive and no comparison between independent groups was planned, no inferential statistical tests or *p*-values were computed.

## 4. Discussion

The present data show that using CAD/CAM nickel–titanium fixed retainers by an experienced operator resulted in a success rate of 77% for the mandibular arch and 78% for the maxillary arch over a 12-month observation period. The most common negative event recorded was wire debonding (19% for the mandibular and 21% for the maxillary arch, respectively), although wire loss was also relatively frequently observed (1% for the mandibular and 2% for the maxillary arch, respectively). In addition, it can be noted that wire breakages remain a relatively rare problem both for maxillary and mandibular retainers over the 12-month observation period. Premolars (when bonded) were the teeth most likely to experience problems with regard to debonding, followed by central incisors.

These results are encouraging, particularly when compared to other studies evaluating similar CAD/CAM retainers. For instance, Jowett et al. [12] reported a failure rate of 50% for Memotain^®^ fixed retainers in the maxillary arch at 6 months, leading to the early discontinuation of their trial. In contrast, Kartal et al. [13] found a 77% survival rate at 6 months for mandibular Memotain^®^ fixed retainers, which was comparable to our 12-month results. Gelin et al. [14] also showed no significant difference between CAD/CAM and conventional stainless-steel retainers in terms of dental stability and retainer survival. Rabia et al. [18] found lower gingival inflammation and calculus accumulation in the Memotain^®^ group compared to stainless steel and dead-soft wires (with no shape memory). Furthermore, looking at the study of Pullisaar and al. [15] comparing two-year stability of CAD/CAM retainers to conventional multistranded fixed retainers, they found no differences in failures and patient satisfaction between groups [15].

When comparing our results with those of traditional multistranded retainers, Renkema et al. [19] reported a failure rate of 32.2% at two years. Their evaluation was based on debonding, breakage, and tooth movement measured using Little’s Irregularity Index, whereas the present study was limited to clinically visible changes. The failure rate was therefore below that reported in the present study for the mandibular arch (23%). Moreover, their failure rate was assessed 2 years after treatment finish, which might explain why it is slightly higher than ours, assessed at 1 year after completing treatment. In addition, their study was somewhat comparable to ours because they calculated the number of failures per tooth type: 3.8% for canines, 13% for lateral incisors, and 14.7% for central incisors at 2 years; while in our study failure rates for the same teeth at 1 year were 2.6%, 3.4%, and 6.8%, respectively.

One possible reason why the success rate per tooth reported in the present study was favourable could be that we used a CAD/CAM fixed retainer that may better be able to respect the lingual anatomy of the teeth. Nonetheless, Renkema and al. [19] focused only on the mandibular arch and thus no comparison could be made with the maxillary arch. Another possible reason explaining different failure rates between studies is the specific bonding method. Renkema and al. [19] did not mention their exact method which may have been different from ours. The bonding method could also be a variable in the success rate of bonded retainers.

In the present study, regarding the mandibular arch, at 6 months the success rate for the central and lateral incisors is lower than for the canines. This slightly lower success rate could be because the bonding surface is smaller on these two types of teeth. At 12 months, the central incisors encounter many debonding problems which seem to be recurrent in some cases. This high debonding rate on central incisors could therefore imply that the surface of the tooth may play a role in the effectiveness of the bonding or that the presence of the transfer key may in some way interfere with the adhesion. Perhaps bonding on to a larger surface of the tooth may be considered to reduce the risk of failure.

One interesting aspect to note was that it was often the same retainers that presented problems both at 6 months as well as at 12 months, which may raise the question of wire-specific or patient-specific factors that can contribute to these problems, or the fact that if a fixed retainer presents problems once, then it may be more likely to present problems a second time. There are however no data to support these hypotheses. In contrast to problems of debonding, few problems of tooth displacement (relapse of tooth position) occurred over the follow-up period which is encouraging since these problems are more complicated to deal with than simply having to rebond a fixed retainer on a specific tooth.

As far as premolars are concerned, the failure rate was rather high, but the smaller number of bonded premolars that were included in the study may make this sample less representative. Nonetheless, the present data suggest that although fixed retainers bonded from premolar to premolar represent 6% of the mandibular retainers placed, they are responsible for 15% of failures. Similarly, for the maxillary arch, fixed retainers bonded from premolar to premolar represent only 5% of the retainers but account for 16% of failures. One should therefore exercise caution when deciding to place fixed retainers extending to the first premolars, with case selection potentially being key. Perhaps to avoid these problems, in clinical situations where it is deemed useful to add the premolars into the fixed retainer, a fixed retainer only to the canines can be prescribed in conjunction with a removable retainer.

The present study has several potential limitations. All fixed retainers were placed by a single experienced operator, which may not reflect general clinical practice and may limit generalisability. Moreover, a substantial number of initially screened patients were excluded due to missing follow-up visits, introducing a potential selection bias toward individuals who are more compliant. Additionally, the sample size for certain subgroups (e.g., fixed retainers extending to premolars) was small, thus reducing the power of the study in this regard. Furthermore, failure assessment was based on clinical observations without standardized indices like Little’s Irregularity Index being used, as the objective of the present study was not to test the ability of the retainer to maintain post-treatment tooth alignment. Finally, as no control group or alternative retainer types were included, comparative effectiveness cannot be inferred.

## 5. Conclusions

At the 12-month follow-up, CAD/CAM nickel–titanium fixed retainers (Memotain^®^) demonstrated almost identical success rates for the mandibular (77%) and maxillary (78%) arches. In the mandibular arch, debonding was the most frequent complication, followed by wire loss and, rarely, breakage. In the maxillary arch, debonding was also the most common event, followed by wire loss, tooth displacement, and breakage. These findings indicate promising clinical performance, with lower mandibular failure rates than those reported in the literature for conventional multistranded retainers, suggesting a potential advantage in enhancing retainer reliability. However, given the divergence in outcomes reported across existing studies, further well-designed, multicentre, and long-term trials are needed to confirm these results and guide clinical recommendations.

## Figures and Tables

**Table 1:** Distribution of the fixed retainers, with number of retainers included.

	Maxillary Arch (*n*)	Mandibular Arch (*n*)	Total (*n* (%))
**Lateral incisor to lateral incisor**	40	-	40 (12%)
**Canine to canine**	114	166	280 (83%)
**Premolar to premolar**	8	10	18 (5%)
**Total (*n* (%))**	**162 (48%)**	**176 (52%)**	**338 (100%)**

**Table 2:** Distribution of debonding events per tooth type in the mandibular arch at 6 months.

	First Premolar	Canine	Lateral Incisor	Central Incisor
**Total number of teeth**	20	352	352	352
**Teeth with no event; number (%)**	20 (100%)	342 (97.2%)	334 (94.9%)	334 (94.9%)
**Teeth with debonding event; number (%)**	0 (0%)	10 (2.8%)	18 (5.1%)	18 (5.1%)

**Table 3:** Distribution of number of events (debonding, wire breakage, wire loss) per tooth type in the mandibular arch at 12 months.

	First Premolar	Canine	Lateral Incisor	Central Incisor
**Total number of teeth**	20	352	352	352
**Teeth with no event; number (%)**	16 (80%)	343 (97.5%)	340 (96.6%)	328 (93.2%)
**Teeth with debonding; number (%)**	1 (5%)	4 (1.1%)	8 (2.3%)	17 (4.8%)
**Teeth with wire breakage; number (%)**	1 (5%)	1 (0.3%)	0 (0%)	3 (0.9%)
**Teeth with wire loss; number (%)**	2 (10%)	4 (1.1%)	4 (1.1%)	4 (1.1%)

**Table 4:** Distribution of number of events (debonding, wire breakage, wire loss, tooth displacement) per tooth type in the maxillary arch at 6 months.

	First Premolar	Canine	Lateral Incisor	Central Incisor
**Total number of teeth**	16	244	324	324
**Teeth with no event; number (%)**	15 (94%)	229 (93.9%)	307 (94.8%)	307 (94.8%)
**Teeth with debonding; number (%)**	1 (6%)	7 (2.9%)	9 (2.8%)	11 (3.4%)
**Teeth with wire breakage; number (%)**	0 (0%)	1 (0.4%)	1 (0.3%)	0 (0%)
**Teeth with wire loss; number (%)**	0 (0%)	2 (0.8%)	2 (0.6%)	2 (0.6%)
**Teeth with displacement; number (%)**	0 (0%)	5 (2.0%)	5 (1.5)	4 (1.2%)

**Table 5:** Distribution of number of events (debonding, wire breakage, wire loss, tooth displacement) per tooth type in the maxillary arch at 12 months.

	First Premolar	Canine	Lateral Incisor	Central Incisor
**Total number of teeth**	16	244	324	324
**Teeth with no event; number (%)**	13 (81.3%)	235 (96.3%)	308 (95.1%)	300 (92.6%)
**Teeth with debonding; number (%)**	1 (6.2%)	2 (0.8%)	9 (2.8%)	17 (5.3%)
**Teeth with wire breakage; number (%)**	0 (0%)	1 (0.4%)	1 (0.3%)	1 (0.3%)
**Teeth with wire loss; number (%)**	2 (12.5%)	6 (2.5%)	6 (1.8%)	6 (1.8%)
**Teeth with displacement; number (%)**	0 (0%)	0 (0%)	0 (0%)	0 (0%)

## Data Availability

The data presented in this study will be made available on reasonable request from the corresponding author.
